# Evaluation of Aspirin and Statin Therapy Use and Adherence in Patients With Premature Atherosclerotic Cardiovascular Disease

**DOI:** 10.1001/jamanetworkopen.2020.11051

**Published:** 2020-08-20

**Authors:** Dhruv Mahtta, David J. Ramsey, Mahmoud Al Rifai, Khurram Nasir, Zainab Samad, David Aguilar, Hani Jneid, Christie M. Ballantyne, Laura A. Petersen, Salim S. Virani

**Affiliations:** 1 Health Policy, Quality and Informatics Program, Health Services Research and Development, Center for Innovations in Quality, Effectiveness, and Safety, Michael E. DeBakey Veterans Affairs Medical Center, Houston, Texas; 2 Section of Health Services Research, Department of Medicine, Baylor College of Medicine, Houston, Texas; 3 Section of Cardiology, Department of Medicine, Baylor College of Medicine, Houston, Texas; 4 Methodist DeBakey Heart and Vascular Center, Houston Methodist Hospital, Houston, Texas; 5 Department of Medicine, The Aga Khan University, Karachi, Pakistan; 6 Division of Cardiology, University of Texas Health Science Center McGovern Medical School, Houston; 7 Section of Cardiology, Michael E. DeBakey Veterans Affairs Medical Center, Houston, Texas; 8 Section of Cardiovascular Research, Department of Medicine, Baylor College of Medicine, Houston, Texas

## Abstract

**Importance:**

Studies on the use of and adherence to secondary prevention therapies in patients with premature and extremely premature atherosclerotic cardiovascular disease (ASCVD) are lacking.

**Objective:**

To evaluate and compare aspirin use, any statin use, high-intensity statin use, and statin adherence among patients with premature or extremely premature ASCVD compared with patients with nonpremature ASCVD.

**Design, Setting, and Participants:**

This multicenter cross-sectional study used the clinical and administrative data sets of the US Department of Veterans Affairs (VA) to identify adult patients with at least 1 primary care visit in the VA health care system between October 1, 2014, and September 30, 2015. The study cohort comprised patients with ASCVD (ischemic heart disease, peripheral arterial disease, or ischemic cerebrovascular disease) who were enrolled in the Veterans With Premature Atherosclerosis (VITAL) registry. Patients with missing data for date of birth or sex and those with limited life expectancy were excluded. Data were analyzed from November 1, 2019, to January 1, 2020.

**Exposures:**

Premature (the first ASCVD event occurred at age <55 years for men and age <65 years for women) vs nonpremature (the first ASCVD event occurred at age ≥55 years for men or age ≥65 years for women) ASCVD and extremely premature (the first ASCVD event occurred at age <40 years) vs nonpremature ASCVD.

**Main Outcomes and Measures:**

The primary outcomes were aspirin use, any statin use, high-intensity statin use, and statin adherence (measured by proportion of days covered [PDC] ≥0.8).

**Results:**

Of the 1 248 158 patients identified, 135 703 (10.9%) had premature ASCVD (mean [SD] age, 49.6 [5.8] years; 116 739 men [86.0%]), 1 112 455 (89.1%) had nonpremature ASCVD (mean [SD] age, 69.6 [8.9] years; 1 104 318 men [99.3%]), and 7716 (0.6%) had extremely premature ASCVD (mean [SD] age, 34.2 [4.3] years; 6576 men [85.2%]). Patients with premature ASCVD vs those with nonpremature ASCVD had lower rates of aspirin use (96 468 [71.1%] vs 860 726 [77.4%]; *P* < .001) and any statin use (98 908 [72.9%] vs 894 931 [80.5%]; *P* < .001); had a statin PDC of 0.8 or higher (57 306 [57.9%] vs 644 357 [72.0%]; *P* < .001); and a higher rate of high-intensity statin use (49 354 [36.4%] vs 332 820 [29.9%]; *P* < .001). Similarly, patients with extremely premature ASCVD were less likely to use aspirin (odds ratio [OR], 0.27; 95% CI, 0.26-0.29), any statin (OR, 0.25; 95% CI, 0.24-0.27), or high-intensity statin (OR, 0.78; 95% CI, 0.74-0.82) and to be statin adherent (OR, 0.44; 95% CI, 0.41-0.47).

**Conclusions and Relevance:**

In this study, patients with premature or extremely premature ASCVD appeared to be less likely to use aspirin or statins and to adhere to statin therapy. This finding warrants further investigation into premature ASCVD and initiatives, including clinician and patient education, to better understand and mitigate the disparities in medication use and adherence.

## Key Points

**Question:**

Are there differences in the aspirin use, statin use, and statin adherence in patients with premature or extremely premature atherosclerotic cardiovascular disease (ASCVD)?

**Findings:**

In this cross-sectional study of 1 248 158 US veterans with ischemic heart disease, peripheral arterial disease, or ischemic cerebrovascular disease, those with premature or extremely premature ASCVD were statistically significantly less likely to receive aspirin or any statin and to adhere to statin therapy.

**Meaning:**

Results of this study suggest that patients with premature or extremely premature ASCVD have a greater accrued lifetime risk of adverse cardiovascular events, which warrant directed initiatives that can mitigate the disparities in medication use and adherence.

## Teaser

This cross-sectional study evaluates the pattern of aspirin use and statin use and adherence in US veterans with premature atherosclerotic cardiovascular disease.

## Introduction

Substantial advancements in prevention of atherosclerotic cardiovascular disease (ASCVD) have led to substantial improvements in adverse cardiovascular events and associated mortality.^1,2,3^ Despite the improvements, the incidence of ASCVD has increased in younger patients.^4^ This trend is prevalent across all 3 domains of ASCVD: ischemic heart disease (IHD), ischemic cerebrovascular disease (ICVD), and peripheral arterial disease (PAD).^5,6,7,8,9,10^ Furthermore, patients with premature ASCVD experience similar rates of all-cause and cardiovascular mortality compared with older adults.^11,12,13,14^

The use of aspirin and high-intensity statin for secondary prevention of ASCVD is well established and endorsed by multisociety guidelines.^15,16,17,18^ A strong association between nonadherence with these secondary prevention measures and increased cardiovascular mortality has also been well demonstrated.^19,20^ Whether the similarity in rates of cardiovascular mortality between older patients with ASCVD and younger patients with premature ASCVD is attributable to less aggressive implementation of and adherence with secondary prevention strategies among younger adults remains unknown because it has not been studied. Although a previous investigation evaluated statin use in younger patients, the study was limited to 1 domain of ASCVD (IHD).^21^ Hence, a thorough assessment of statin use across the entire spectrum of patients with premature ASCVD is unavailable. Data on statin adherence and the use of aspirin in this population are also scarce.

The primary objective of this cross-sectional study was to evaluate aspirin use, statin use, and statin adherence in patients with premature ASCVD vs in patients with nonpremature ASCVD. We also investigated similar outcomes among patients with extremely premature ASCVD because these individuals are at risk for a higher accrued lifetime morbidity and health care financial burden.

## Methods

The study protocol was approved, and informed consent was waived by the institutional review board at Baylor College of Medicine. The data obtained for this study did not involve interaction with any patients and did not include any individually identifiable information. We followed the Strengthening the Reporting of Observational Studies in Epidemiology (STROBE) reporting guideline.^22^

Using the US Department of Veterans Affairs (VA) clinical and administrative data sets, we identified 1 248 158 patients with ASCVD aged 18 years or older who received primary care services between October 1, 2014, and September 30, 2015 (VA fiscal year 2015), across the 130 main VA facilities and their associated community-based outpatient clinics in the US. The most recent primary care visit during fiscal year 2015 was used as the index primary care physician (PCP) visit and as an anchor for the present analyses. Additional details regarding this cohort have been described previously.^23^ The presence of ASCVD was defined as a history of IHD, ICVD, or PAD, which was ascertained from recorded *International Classification of Diseases, Ninth Revision, Clinical Modification* diagnosis and procedural codes or *Current Procedural Terminology* codes.^24,25^ Based on a manual medical record review of 200 patients, the algorithm we used computed a 95% positive predictive value for correct identification of ASCVD.^26,27^

We analyzed patients enrolled in the nationwide Veterans With Premature Atherosclerosis (VITAL) registry, which was created from the large cohort of adults with ASCVD.^28^ In accordance with national cardiovascular guidelines, premature ASCVD was defined as the first ASCVD event occurring before age 55 years for men and before age 65 years for women.^29^ These adults with premature ASCVD were included in the VITAL registry. Nonpremature ASCVD was defined as the first ASCVD event occurring at age 55 years or older for men or age 65 years or older for women. Furthermore, extremely premature ASCVD was defined as the first ASCVD event occurring before age 40 years. Patients with nonpremature and extremely premature ASCVD were also included in this study. Aside from patients with missing date of birth or sex data, patients with limited life expectancy, as indicated by a history of metastatic cancer in the past 5 years or receipt of hospice care in the past 12 months, were excluded from the analyses.^30^

Clinical data sources in the VA system were used to identify patient age, sex, race/ethnicity, and body mass index (calculated as weight in kilograms divided by height in meters squared). Medical history of hypertension, diabetes, myocardial infarction, IHD, ICVD, and PAD was ascertained from *International Classification of Diseases, Ninth Revision, Clinical Modification* and *Current Procedural Terminology* codes. The VA data sets included baseline levels of low-density lipoprotein cholesterol, non–high-density lipoprotein cholesterol, triglycerides, lipoprotein A, and apolipoprotein B. The Diagnosis Cost Group (DCG) relative risk score, a well-established and validated surrogate marker of the overall illness burden, was also calculated.^31,32^ A DCG relative risk score of 1 signified an average illness burden; a score above 1, a higher-than-average illness burden; and a score below 1, a lower-than-average illness burden. We also identified various facility-level and clinician-level variables, including receipt of care from a physician vs an advanced practice practitioner (nurse practitioner or physician assistant), a teaching vs nonteaching facility, and an urban vs a rural facility; median number of PCP visits in the 12 months before the index PCP visit; and percentage of patients receiving cardiological and PCP services in the 12 months before the index PCP visit. We ascertained the median number of days from the first ASCVD event to the index PCP visit, nonaspirin antiplatelet use, and anticoagulant use among this cohort.

The primary outcomes in this study were aspirin use, any statin use, high-intensity statin use, and statin adherence, which were ascertained from the VA pharmacy data. The term *use* referred to an existing prescription for the said medication within 100 days before or 14 days after the index PCP visit. The prescribed statin drugs included atorvastatin, rosuvastatin, pravastatin, fluvastatin, lovastatin, simvastatin, and pitavastatin. In accordance with the national cholesterol guidelines,^33^ high-intensity statin was defined as atorvastatin, 40 mg or more, or rosuvastatin, 20 mg or more. We included both aspirin and statin therapy that were prescribed outside the VA health care system if proper notations were made in the VA primary care encounter. Adherence to statin therapy was assessed by the proportion of days covered (PDC), a well-established and validated method that has been documented previously.^32,34^ The PDC was calculated by dividing the number of days with (covered by) a certain statin prescription refill by the total number of days within that period.^35^ The PDC calculations accounted for changes in statin doses or type of statin and early refills. If statin dose was changed, we assumed pill splitting or doubling to match the new dose from the previous supply. If the statin type was changed, we assumed the remaining supply of the previous statin was discarded in favor of the new statin. We also accounted for stockpiling by assuming that early refills were not consumed until the previous supply was finished. Based on data from previous studies on medication adherence,^36^ patients were deemed to be adherent to statin therapy if the PDC was 0.8 or higher. We measured the PDC both as a categorical variable (≥0.8 or <0.8) and as a continuous variable. The term *adherence* referred to the filling or refilling of prescriptions by the patients.

### Statistical Analysis

We assessed the distribution of various patient-, facility-, and clinician-level variables across patients with premature and nonpremature ASCVD. Categorical variables were analyzed with a χ^2^ test, and continuous variables were analyzed with an unpaired, 2-tailed *t* test. Two-sided *P* < .05 was used to indicate statistical significance.

Use of aspirin, any statin, and high-intensity statin and statin adherence were evaluated across patients with premature ASCVD and nonpremature ASCVD. Subsequently, we created multivariable hierarchical logistic and linear regression models to study the association between premature ASCVD and aspirin use, any statin use, high-intensity statin use, and statin adherence. Identical analytical methods were used to study patients with extremely premature ASCVD. The referent category included patients with nonpremature ASCVD.

All analyses were adjusted for sex, race/ethnicity, type of ASCVD, history of obesity, hypertension, diabetes, DCG relative risk score, median number of days from first ASCVD event to index PCP visit, and facility- and clinician-level covariates. The regression models for aspirin use were adjusted for nonaspirin antiplatelet use and anticoagulant use. Generalized linear latent and mixed models adjusted the regression models for facility-level clustering of patients. Analyses were performed with SAS, version 9.1.3 (SAS Institute Inc) and Stata, version 14 (StataCorp LLC) from November 1, 2019, to January 1, 2020.

## Results

A total of 1 248 158 patients with ASCVD were identified after excluding patients with missing date of birth or sex data (n = 95), with limited life expectancy (n = 28 316), or with missing DCG relative risk score variable (n = 129). After age-based exclusion was applied, a total of 135 703 patients (10.9%) with premature ASCVD (mean [SD] age, 49.6 [5.8] years; 116 739 men [86.0%]) were included in the VITAL registry (Table 1). Accordingly, 1 112 455 patients (89.1%) were identified as having nonpremature ASCVD (mean [SD] age, 69.6 [8.9] years; 1 104 318 men [99.3%]).

**Table 1.** Baseline Characteristics of Patients With or Without Premature ASCVD

Characteristic	No. (%)		*P* value
	Patients with premature ASCVD (n = 135 703)^**a**^	Patients with nonpremature ASCVD (n = 1 112 455)^**b**^	
Demographic			
Age, mean (SD), y	49.6 (5.8)	69.6 (8.9)	<.001
Male sex	116 739 (86.0)	1 104 318 (99.3)	<.001
Race/ethnicity			
Asian	967 (0.7)	4898 (0.4)	<.001
Black	34 008 (25.1)	110 516 (9.9)	<.001
White	90 835 (66.9)	891 800 (80.2)	<.001
Medical history			
BMI ≥30	75 519 (55.7)	442 891 (39.8)	<.001
Hypertension	127 519 (94.0)	1 064 670 (95.7)	<.001
Diabetes	65 263 (48.1)	565 444 (50.8)	<.001
IHD	105 659 (77.9)	884 063 (79.5)	<.001
Myocardial infarction	64 371 (47.4)	278 025 (25.0)	<.001
ICVD	38 275 (28.2)	311 168 (27.9)	.07
PAD	16 890 (12.5)	178 859 (16.1)	<.001
LDL-C, mean (SD), mg/dL	150.84 (44.04)	134.02 (40.46)	<.001
Non–HDL-C, mean (SD), mg/dL	193.16 (61.45)	168.73 (50.52)	<.001
Total cholesterol, mean (SD), mg/dL	238.89 (63.65)	214.20 (51.62)	<.001
Lp(a), mean (SD), mg/dL	40.49 (58.32)	31.42 (42.71)	<.001
apoB, mean (SD), mg/dL	106.26 (37.09)	94.02 (31.39)	<.001
Overall health status and health care use			
No. of days from ASCVD event to index PCP visit, median (IQR)	1658 (1001-3002)	1594 (1069-1780)	<.001
DCG relative risk score, mean (SD)	2.18 (2.59)	1.54 (2.13)	<.001
Nonaspirin antiplatelet use	29 948 (22.1)	268 270 (24.1)	<.001
Anticoagulant use	14 196 (10.5)	188 459 (16.9)	<.001
Facility- and clinician-level characteristics			
Receiving care at teaching facility	63 360 (46.7)	421 523 (37.9)	<.001
PCP	105 255 (77.6)	855 134 (76.9)	<.001
Receiving care at rural facility	9643 (7.1)	86 047 (7.7)	<.001
Patients with a PCP visit in the 12 mo before index PCP visit	128 781 (94.9)	1 018 122 (91.5)	<.001
Patients with a cardiology visit in the 12 mo before index PCP visit	36 750 (27.1)	228 154 (20.5)	<.001
No. of PCP visits in the 12 mo before index PCP visit, median (IQR)	4 (2-7)	3 (1-6)	<.001

Abbreviations: apoB, apolipoprotein B; ASCVD, atherosclerotic cardiovascular disease; BMI, body mass index (calculated as weight in kilograms divided by height in meters squared); DCG, Diagnostic Cost Group; HDL-C, high-density lipoprotein cholesterol; ICVD, ischemic cerebrovascular disease; IHD, ischemic heart disease; IQR, interquartile range; LDL-C, low-density lipoprotein cholesterol; Lp(a), lipoprotein A; PAD, peripheral arterial disease; PCP, primary care physician.

SI conversion factors: To convert apoB to grams per liter, multiply by 0.01; HDL-C, LDL-C, and total cholesterol to millimoles per liter, multiply by 0.0259; and Lp(a) to milligrams per liter, multiply by 0.1.

^a^ Patients with premature ASCVD were those who experienced their first ASCVD event before age 55 years for men and before age 65 years for women.

^b^ Patients with nonpremature ASCVD were those who experienced their first ASCVD event at age 55 years or older for men and age 65 years or older for women.

The premature ASCVD group compared with the nonpremature ASCVD group comprised a higher proportion of female (18 964 [14.0%] vs 8137 [0.7%]), Asian (967 [0.7%] vs 4898 [0.4%]), and Black patients (34 008 [25.1%] vs 110 516 [9.9%]) and a lower proportion of White patients (90 835 [66.9%] vs 891 800 [80.2%]). A higher proportion of patients with premature ASCVD vs those with nonpremature ASCVD had a body mass index of 30 or higher (75 519 of 135 703 [55.7%] vs 442 891 of 1 112 455 [39.8%]), whereas a lower proportion had a history of diabetes (65 263 [48.1%] vs 565 444 [50.8%]) and hypertension (127 519 [94.0%] vs 1 064 670 [95.7%]). The prevalence of IHD (105 659 [77.9%] vs 884 063 [79.5%]) and PAD (16 890 [12.5%] vs 178 859 [16.1%]) was lower and the prevalence of myocardial infarction was higher (64 371 [47.4%] vs 278 025 [25.0%]) among patients with premature ASCVD than in those with nonpremature ASCVD. Patients with premature ASCVD had higher levels of mean (SD) total cholesterol (238.89 [63.65] mg/dL vs 214.20 [51.62] mg/dL), low-density lipoprotein cholesterol (150.84 [44.04] mg/dL vs 134.02 [40.46] mg/dL [to convert cholesterol levels to millimoles per liter, multiply by 0.0259]), and non–high-density lipoprotein cholesterol (193.16 [61.45] mg/dL vs 168.73 [50.52] mg/dL) (*P* < .001 for all comparisons).

Table 2 shows that a statistically significantly lower proportion of patients with premature ASCVD vs nonpremature ASCVD received aspirin therapy (96 468 [71.1%] vs 860 726 [77.4%]; *P* < .001) and any statin therapy (98 908 [72.9%] vs 894 931 [80.5%]; *P* < .001). However, a higher number of patients with premature ASCVD received high-intensity statin (49 354 [36.4%] vs 332 820 [29.9%]; *P* < .001). Patients with premature ASCVD had a statistically significantly lower mean (SD) PDC (0.71 [0.32] vs 0.80 [0.29]; adjusted odds ratio [OR], −0.083 [95% CI, −0.084 to −0.081]; *P* < .001) and a lower proportion of patients with PDC of 0.8 or higher (57 306 [57.9%] vs 644 357 [72.0%]; *P* < .001). In fully adjusted regression models, premature ASCVD was associated with a lower likelihood of aspirin use (OR, 0.69; 95% CI, 0.68-0.70), any statin use (OR, 0.70; 95% CI, 0.69-0.71), and statin adherence (OR, 0.56; 95% CI, 0.55-0.57) (Figure). In contrast, premature ASCVD was independently associated with a 37% higher likelihood of high-intensity statin use (OR, 1.37; 95% CI, 1.35-1.39).

**Table 2.** Aspirin Use, Statin Use, and Statin Adherence Among Patients With Premature ASCVD

Variable	No. (%)		Adjusted OR or β coefficient (95% CI)^**c**^	*P* value
	Patients with premature ASCVD (n = 135 703)^**a**^	Patients with nonpremature ASCVD (n = 1 112 455)^**b**^		
Aspirin use^d^	96 468 (71.1)	860 726 (77.4)	0.69 (0.68 to 0.70)^d^	<.001
Statin use				
Any	98 908 (72.9)	894 931 (80.5)	0.70 (0.69 to 0.71)^c^	<.001
High-intensity	49 354 (36.4)	332 820 (29.9)	1.37 (1.35 to 1.39)^c^	<.001
Statin PDC				
≥0.8	57 306 (57.9)	644 357 (72.0)	0.56 (0.55 to 0.57)^c^	<.001
Mean (SD)	0.71 (0.32)	0.80 (0.29)	−0.083 (−0.084 to −0.081)	<.001

Abbreviations: ASCVD, atherosclerotic cardiovascular disease; OR, odds ratio; PDC, proportion of days covered.

^a^ Patients with premature ASCVD were those who experienced their first ASCVD event before age 55 years for men and before age 65 years for women.

^b^ Patients with nonpremature ASCVD were those who experienced their first ASCVD event at age 55 years or older for men and age 65 years or older for women.

^c^ Adjusted for sex, race/ethnicity, obesity (body mass index ≥30 [calculated as weight in kilograms divided by height in meters squared]), hypertension, diabetes, type of ASCVD (ischemic heart disease vs peripheral arterial disease vs ischemic cerebrovascular disease), clinician type (physician vs advanced practice practitioner), teaching vs nonteaching facility, urban vs rural facility, number of cardiology visits 12 months before index primary care physician (PCP) visit, median number of PCP visits 12 months before index PCP visit, median number of days from first ASCVD event to the index PCP visit, and Diagnostic Cost Group relative risk score.

^d^ Regression model for aspirin use was adjusted for nonaspirin platelet use and anticoagulant use in addition to all of the aforementioned covariates.

**Figure.**
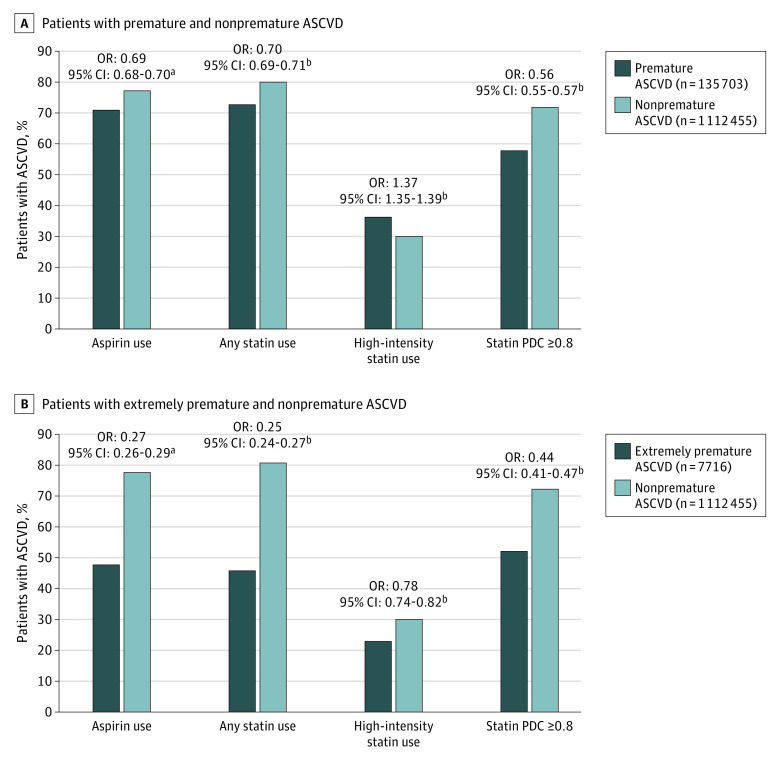
Rates of Use and Adherence With Statin and Aspirin Therapy Among Patients With Premature and Extremely Premature Atherosclerotic Cardiovascular Disease (ASCVD) Adjusted odds ratios (ORs) represent independent odds of medication use or medication adherence among patients with either premature ASCVD or extremely premature ASCVD compared with patients with nonpremature ASCVD. The true OR has a 95% certainty of falling between the specified CI range. PDC indicates the proportion of days covered. ^a^Aspirin use regression models were adjusted for nonaspirin antiplatelet use and anticoagulant use in addition to sex, race/ethnicity, ischemic heart disease (IHD), obesity (body mass index [BMI] ≥30 [calculated as weight in kilograms divided by height in meters squared]), hypertension, diabetes, clinician type (physician vs advanced practice practitioner [APP]), teaching vs nonteaching facility, urban vs rural facility, number of cardiology visits 12 months before the index primary care physician (PCP) visit, median number of PCP visits 12 months before the index PCP visit, median number of days from first ASCVD event to the index PCP visit, and Diagnostic Cost Group (DCG) relative risk score (marker of the overall illness burden of the patient). ^b^Adjusted for sex, race/ethnicity, IHD, obesity (BMI ≥30), hypertension, diabetes, clinician type (physician vs APP), teaching vs nonteaching facility, urban vs rural facility, number of cardiology visits 12 months before the index PCP visit, median number of PCP visits 12 months before the index PCP visit, median number of days from first ASCVD event to the index PCP visit, and DCG relative risk score.

A total of 7716 patients (0.6%) with extremely premature ASCVD (mean [SD] age, 34.2 [4.3] years; 6576 men [85.2%] and 1140 women [14.8%]) were identified (Table 3). Compared with patients with nonpremature ASCVD, patients with extremely premature ASCVD had a higher proportion of female (1140 [14.8%] vs 8137 [0.7%]), Asian (111 [1.4%] vs 4898 [0.4%]), and Black patients (1859 [24.1%] vs 110 516 [9.9%]) and a lower proportion of White patients (5171 [67.0%] vs 891 800 [80.2%]). A statistically significantly higher proportion of patients with extremely premature ASCVD had a body mass index of 30 or higher (75 519 of 135 703 [55.7%] vs 442 891 of 1 112 455 [39.8%]) and had a history of myocardial infarction (3534 [45.8%] vs 278 025 [25.0%]). However, the prevalence of hypertension (6334 [82.1%] vs 1 064 670 [95.7%]) and diabetes (1960 [25.4%] vs 565 444 [50.8%]) was lower. A higher prevalence of ICVD (2499 [32.4%] vs 311 168 [28.0%]) and a lower prevalence of IHD (5180 [67.1%] vs 884 063 [79.5%]) and PAD (587 [7.6%] vs 178 859 [16.1%]) were observed among patients with extremely premature ASCVD. In addition, these patients were observed to have higher mean (SD) levels of total cholesterol (229.51 [64.36] mg/dL vs 214.20 [51.62] mg/dL), low-density lipoprotein cholesterol (145.67 [46.87] mg/dL vs 134.02 [40.46] mg/dL), and non–high-density lipoprotein cholesterol (184.88 [63.54] mg/dL vs 168.73 [50.52] mg/dL) (*P* < .001 for all comparisons).

**Table 3.** Baseline Characteristics of Patients With or Without Extremely Premature ASCVD

Characteristic	No. (%)		*P* value
	Patients with extremely premature ASCVD (n = 7716)^**a**^	Patients with nonpremature ASCVD (n = 1 112 455)^**b**^	
Demographic			
Age, mean (SD), y	34.2 (4.3)	69.6 (8.9)	<.001
Male sex	6576 (85.2)	1 104 318 (99.3)	<.001
Race/ethnicity			
Asian	111 (1.4)	4898 (0.4)	<.001
Black	1859 (24.1)	110 516 (9.9)	<.001
White	5171 (67.0)	891 800 (80.2)	<.001
Medical history			
BMI ≥30	4407 (57.3)	442 891 (39.9)	<.001
Hypertension	6334 (82.1)	1 064 670 (95.7)	<.001
Diabetes	1960 (25.4)	565 444 (50.8)	<.001
IHD	5180 (67.1)	884 063 (79.5)	<.001
Myocardial infarction	3534 (45.8)	278 025 (25.0)	<.001
ICVD	2499 (32.4)	311 168 (28.0)	<.001
PAD	587 (7.6)	178 859 (16.1)	<.001
LDL-C, mean (SD), mg/dL	145.67 (46.87)	134.02 (40.46)	<.001
Non–HDL-C, mean (SD), mg/dL	184.88 (63.54)	168.73 (50.52)	<.001
Total cholesterol, mean (SD), mg/dL	229.51 (64.36)	214.20 (51.62)	<.001
Lp(a), mean (SD), mg/dL	43.34 (42.73)	31.42 (42.71)	.15
apoB, mean (SD), mg/dL	122.45 (33.17)	94.02 (31.39)	<.001
Overall health status and health care use			
No. of days from ASCVD event to index PCP visit, median (IQR)	1395 (668-2099)	1594 (1069-1780)	<.001
DCG relative risk score, mean (SD)	1.95 (2.38)	1.54 (2.13)	<.001
Nonaspirin antiplatelet use	1037 (13.4)	268 270 (24.1)	<.001
Anticoagulant use	754 (9.8)	188 459 (16.9)	<.001
Facility- and clinician-level characteristics			
Receiving care at teaching facility	3701 (48.0)	421 523 (37.9)	<.001
PCP	5866 (76.0)	855 134 (76.9)	.08
Receiving care at rural facility	499 (6.5)	86 047 (7.7)	<.001
Patients with a PCP visit in the 12 mo before index PCP visit	7155 (92.7)	1 018 122 (91.5)	<.001
Patients with a cardiology visit in the 12 mo before index PCP visit	1671 (21.7)	228 154 (20.5)	.01
No. of PCP visits in the 12 mo before index PCP visit, median (IQR)	4 (2-6)	3 (1-6)	<.001

Abbreviations: apoB, apolipoprotein B; ASCVD, atherosclerotic cardiovascular disease; BMI, body mass index (calculated as weight in kilograms divided by height in meters squared); DCG, Diagnostic Cost Group; HDL-C, high-density lipoprotein cholesterol; ICVD, ischemic cerebrovascular disease; IHD, ischemic heart disease; IQR, interquartile range; LDL-C, low-density lipoprotein cholesterol; Lp(a), lipoprotein A; PAD, peripheral arterial disease; PCP, primary care physician.

SI conversion factors: To convert apoB to grams per liter, multiply by 0.01; HDL-C, LDL-C, and total cholesterol to millimoles per liter, multiply by 0.0259; and Lp(a) to milligrams per liter, multiply by 0.1.

^a^ Patients with extremely premature ASCVD were those who experienced their first ASCVD event before age 40 years.

^b^ Patients with nonpremature ASCVD were those who experienced their first ASCVD event at age 55 years or older for men and age 65 years or older for women.

A lower proportion of patients with extremely premature ASCVD vs those with nonpremature ASCVD received aspirin (3668 [47.5%] vs 860 726 [77.4%]; *P* < .001), any statin (3523 [45.7%] vs 894 931 [80.5%]; *P* < .001), and high-intensity statin (1755 [22.7%] vs 332 820 [29.9%]; *P* < .001) (Table 4). The mean (SD) statin PDC (0.64 [0.36] vs 0.80 [0.29]; adjusted OR, −0.15 [95% CI, −0.16 to −0.14]; *P* < .001) and proportion of patients with statin PDC of 0.8 or higher (1830 [51.9%] vs 644 357 [72.0%]; *P* < .001) were lower among patients with extremely premature ASCVD. Extremely premature ASCVD was independently associated with lower odds of aspirin use (OR, 0.27; 95% CI, 0.26-0.29), any statin use (OR, 0.25; 95% CI, 0.24-0.27), and high-intensity statin use (OR, 0.78; 95% CI, 0.74-0.82) (Figure). The presence of extremely premature ASCVD was also associated with a 56% lower likelihood of statin adherence (OR, 0.44; 95% CI, 0.41-0.47).

**Table 4.** Aspirin Use, Statin Use, and Statin Adherence Among Patients With Extremely Premature Atherosclerotic Cardiovascular Disease

Variable	No. (%)		Adjusted OR/β coefficient (95% CI)^**c**^	*P* value
	Patients with extremely premature ASCVD (n = 7716)^**a**^	Patients with nonpremature ASCVD (n = 1 112 455)^**b**^		
Aspirin use^d^	3668 (47.5)	860 726 (77.4)	0.27 (0.26 to 0.29)^d^	<.001
Statin use				
Any	3523 (45.7)	894 931 (80.5)	0.25 (0.24 to 0.27)^c^	<.001
High-intensity	1755 (22.7)	332 820 (29.9)	0.78 (0.74 to 0.82)^c^	<.001
Statin PDC				
≥0.8	1830 (51.9)	644 357 (72.0)	0.44 (0.41 to 0.47)^c^	<.001
Mean (SD)	0.64 (0.36)	0.80 (0.29)	−0.15 (−0.16 to −0.14)	<.001

Abbreviations: ASCVD, atherosclerotic cardiovascular disease; OR, odds ratio; PDC, proportion of days covered.

^a^ Patients with extremely premature ASCVD were those who experienced their first ASCVD event before age 40 years.

^b^ Patients with nonpremature ASCVD were those who experienced their first ASCVD event at age 55 years or older for men and age 65 years or older for women.

^c^ Adjusted for sex, race/ethnicity, obesity (body mass index ≥30 [calculated as weight in kilograms divided by height in meters squared]), hypertension, diabetes, type of ASCVD (ischemic heart disease vs peripheral arterial disease vs ischemic cerebrovascular disease), clinician type (physician vs advanced practice practitioner), teaching vs nonteaching facility, urban vs rural facility, number of cardiology visits 12 months before index primary care physician (PCP) visit, median number of PCP visits 12 months before index PCP visit, median number of days from first ASCVD event to the index PCP visit, and Diagnostic Cost Group relative risk score.

^d^ Regression model for aspirin use was adjusted for nonaspirin platelet use and anticoagulant use in addition to all of the aforementioned covariates.

## Discussion

This study demonstrated that patients with premature ASCVD or extremely premature ASCVD were less likely to receive aspirin and any statin therapy than patients with nonpremature ASCVD. Patients with premature ASCVD were more likely to receive guideline-concordant high-intensity statin, whereas those with extremely premature ASCVD were less likely to receive this medication. Furthermore, this study showed that all patients, regardless of their age of ASCVD onset, had suboptimal use of these secondary prevention medications. Presence of premature or extremely premature ASCVD was independently associated with lower statin adherence. Such differences between groups were evident despite patients with premature or extremely premature ASCVD having a higher frequency of outpatient encounters with PCPs and cardiovascular specialists than patients with nonpremature ASCVD.

These results present evidence of the existing knowledge gap regarding aspirin and statin use for secondary prevention among patients with premature ASCVD. The rationale behind the observed disparities in prescription patterns is likely multifactorial. Although accurate, the prevailing emphasis on older age as a leading risk factor of ASCVD^37,38,39^ may create the misperception that younger age has protective properties against ASCVD. Furthermore, as observed in this study, the prevalence of metabolic risk factors, such as hypertension and diabetes, was often lower among patients with premature or extremely premature ASCVD. Therefore, it may be hypothesized that in such patients, nontraditional and nonmetabolic risk factors (eg, hereditary thrombophilia, inflammatory disorders) may instead be more prevalent.^40,41^ The treatment of nontraditional and nonmetabolic conditions may supersede the secondary prevention of ASCVD. In patients with premature ASCVD, the occurrence of the index ASCVD event at a younger age may be incorrectly perceived as an isolated episode rather than as the true risk for recurrent adverse cardiovascular events.

Therapeutic inertia, which is defined as the lack of initiation or intensification of clinically indicated treatment,^42,43^ may also be an important factor in these observed differences. The prevalence of therapeutic inertia in statin initiation may be high among patients with premature or extremely premature ASCVD despite their less favorable baseline lipid profiles. However, the discordance observed in high-intensity statin use between patients with premature ASCVD and those with extremely premature ASCVD may indicate a higher prevalence of therapeutic inertia in statin intensification among patients with extremely premature ASCVD (age of ASCVD onset, 18-39 years) than among patients with premature ASCVD (age of ASCVD onset, 40-54 years for men and 40-64 years for women). Patients with extremely premature ASCVD may experience lower rates of statin therapy intensification because of preconceived notions that these patients do not have as high a risk or do not need high doses at a young age. Such patient-level or clinician-level misconceptions may impede statin intensification among patients with extremely premature ASCVD.

The presence of premature or extremely premature ASCVD was also associated with lower statin adherence. This finding is consistent with the finding in a recent investigation that individuals younger than 55 years were less likely to be statin adherent after a myocardial infarction.^44^ Younger patients with premature or extremely premature ASCVD may exhibit unrealistic optimism bias^45,46^ and lack the necessary understanding of their disease process to foster optimal statin adherence. Similarly, an overall higher functional status after the index ASCVD event may be misinterpreted by patients as indicating their reduced need for statin therapy. Although clinical evidence of statin-associated adverse effects based on patient age is lacking,^47^ the perceived statin-associated adverse effects among the premature ASCVD population may be another factor in statin discontinuation and lower adherence rates. As previously reported, social determinants, such as health literacy and social support, may be associated with medication adherence.^48^ Hence, in the present study, the suboptimal adherence observed among patients with premature or extremely premature ASCVD may be attributed in part to socioeconomic factors. Statin misinformation or misconceptions because of a greater reliance on social media–based or non–clinician-based resources and a comparative lack of experience in long-term medication management^49^ may contribute to lower rates of statin adherence in patients, especially in those with premature ASCVD.

To our knowledge, this study was the first to assess the nationwide disparities in aspirin use, statin use, and statin adherence among US veterans with premature or extremely premature ASCVD. The findings revealed the extent of underuse of and suboptimal adherence to secondary prevention medications in this younger but at-risk population despite adequate clinical encounters with PCPs and cardiovascular specialists. Suboptimal secondary preventive measures in patients with premature ASCVD may be associated with a higher accrued lifetime morbidity risk and financial burden on the health care system. Hence, it is imperative for investigators and health care policy advisors to recognize the presence and implications of the disparities in medication use and adherence. Future efforts are needed to provide clinician and patient education to discredit misperceptions, conduct qualitative and outcomes research into premature ASCVD, and implement research findings. In addition, evidence-based strategies, such as motivational interviewing and the Screening, Brief Intervention and Referral to Treatment approach, may be adopted by clinicians to partially overcome some of the socioeconomic barriers to medication use and adherence.

### Limitations

This study has some limitations. Because of the inherent constraints of clinical data sets and the observational design of the study, we were unable to ascertain and adjust for additional confounders, such as health literacy of patients, the presence of polypharmacy, and the presence of statin-associated adverse effects or aspirin allergy. We were unable to integrate data from non-VA pharmacies to identify additional medication filled outside of the VA system, including over-the-counter aspirin use. Although the VA health care system serves a preponderance of older patients with nonpremature ASCVD, the number of younger and female patients with premature ASCVD included in the VITAL registry remained substantially large given the national scope of the data set. The generalizability of the findings to other non-VA facilities may be imperfect.

## Conclusions

In this cross-sectional study, the presence of premature ASCVD appeared to be associated with a lower likelihood of aspirin use, any statin use, and statin adherence but a higher likelihood of high-intensity statin use in contrast to nonpremature ASCVD. Independent associations were observed between the presence of extremely premature ASCVD and lower odds of aspirin use, any statin use, high-intensity statin use, and statin adherence. Despite guideline recommendations, secondary prevention use of aspirin and statins remained suboptimal in younger patients with premature or extremely premature ASCVD. Further research into premature ASCVD, clinician and patient education, and policy implementation are necessary to better comprehend and mitigate the disparities in medication use and adherence.
